# Gender imbalance in executive management positions at the Mexican National Institutes of Health

**DOI:** 10.1186/s12960-020-0463-4

**Published:** 2020-03-18

**Authors:** Lucero Soledad Rivera-Romano, Cristobal Fresno, Enrique Hernández-Lemus, Mireya Martínez-García, Maite Vallejo

**Affiliations:** 1 National Institute of Cardiology “Ignacio Chavez”, Juan Badiano 1, Mexico City, Mexico; 2 National Institute of Genomic Medicine, Periferico Sur 4809, Mexico City, Mexico; 3 National Institute of Genomic Medicine, Periferico Sur 4809, Mexico City, Mexico

**Keywords:** Gender bias, Women’s rights, Healthcare leadership, Glass ceiling, Gender pay gap, Health workforce

## Abstract

**Background:**

Around the world, there is a significant difference in the proportion of women with access to leadership in healthcare with respect to men. This article studies gender imbalance and wage gap in managerial, executive, and directive job positions at the Mexican National Institutes of Health.

**Methods:**

Cohort data were described using a visual circular representation and modeled using a generalized linear model. Analysis of variance was used to assess model significance, and posterior Fisher’s least significant differences were analyzed when appropriate.

**Results:**

This study demonstrated that there is a gender imbalance distribution among the hierarchical position at the Mexican National Health Institutes and also exposed that the wage gap exists mainly in the (highest or lowest) ranks in hierarchical order.

**Conclusions:**

Since the majority of the healthcare workforce is female, Mexican women are still underrepresented in executive and directive management positions at national healthcare organizations.

## Background

Around the world, women constitute almost 78% of the health workforce; however, most of the female healthcare-related positions belong to the operating level [1–3].

This problem is not restricted to medicine. This phenomenon is common in science, technology, engineering, and mathematics (STEM) fields. A recent survey in the United States of America identified STEM as one of the most male-dominated working environments, even though women percentage in executive positions there increased from 13 to 36% over an 8-year span [4].

Men in holding top management positions continue to emerge as the executive manager figure, more often than women, 44% and 27%, respectively [5]. As estimated by Bertrand and Hallock [6], between the 1990s and the 2000s, women have experienced a somewhat large increase (400%) in their representation in executive positions (from 1.3% in 1992 to around 6.7% in 2004), but the gender gap remains large [7]. This so-called gender wage gap is the difference between the average net pay of men and women for carrying out work of equal or comparable work activities [8]. The World Economic Forum estimated that the global gender economical wage gap would take the next 217 years to close! [9].

It is thus a somehow established fact that women are underrepresented in governance and leadership positions in healthcare and scientific disciplines, all over the world [10, 11]. In what follows, we will sketch what is the current situation in elite Mexican healthcare and biomedical research institutions.

### The Mexican scenario

Women constitute around 51% of Mexico’s population [8]. According to the National Institute of Statistics, Geography and Informatics (Instituto Nacional de Estadística, Geografía e Informática, INEGI), among more than 29 million employed persons reported by the Economic Census of 2014, women reached 43.8% [12].

On the other hand, income inequalities are a well-known situation in relation to female Mexican workers [13, 14]. Regarding the wage gap, the National Council for the Prevention of Discrimination’s (Consejo Nacional para Prevenir la Discriminación, 2017) report informed that women earn 34.2% less than men in the same type of job [15].

A complementary study found out that under equal working characteristics, women earn 12.4% less wages than men [16]. The Employment Outlook study 2016, developed by the Organization for Economic Cooperation and Development (OECD), pointed out that in the last decade, the gender wage gap in Mexico went from 17 to 18% and remains above the OECD average, which was 15% in that period. This gender wage gap in Mexico means that a woman must work around 15 months to earn the same as a man receives in 12 [8].

Currently, the number of female college students exceeds one million and its proportion has become almost equal, since out of every 100 students, 50 are women [17].

However, in the healthcare work field, most leaders and executive positions in healthcare environments are reserved for men [18, 19]; as a result, the critical mass of women in executive management positions has not been reached. In this way, the impact of institutional medical policies on the gender perspective has been delayed [20].

### The Mexican National Institutes of Health

In Mexico, the modern public health was born at the beginning of the twentieth century with the foundation of the first nationwide medical center-level hospital, *El hospital general de Mexico*, that was to become the cradle of several medical speciality centers and of the Mexican National Institutes of Health (MNIHs) [21].

In 1943, the Mexican Health Ministry (then called Secretaria de Salubridad y Asistencia, now Secretaria de Salud (SSA)) was established as a federal organism to regulate, administer, and promote public health. During that same year, the first National Institute of Health (MNIH), the Hospital Infantil de Mexico Federico Gomez, was founded [22].

One year later (1944), the second MNIH was founded, the Instituto Nacional de Cardiología, and in 1946, the third one, El Hospital de Enfermedades de la Nutrición (Now Instituto Nacional de Ciencias Médicas y Nutrición Salvador Zubirán). In time, ten more MNIHs were created, and finally, the latest addition to the MNIHs is the Instituto Nacional de Geriatría (see Table 1) [21].

**Table 1:** Mexican National Institutes of Health (MNIHs)

MNIH	Year of	Medical	Acronyms	Female
	foundation	speciality		general direction
Hospital Infantil de México				
*Federico Gómez*	1943	Pediatrics	HINFG	Never
Instituto Nacional de Cardiología				
*Ignacio Chávez*	1944	Cardiology	INCICH	Never
Instituto Nacional de Ciencias Médicas y Nutrición				
*Salvador Zubirán*	1946	Internal medicine	INCMNSZ	Never
Instituto Nacional de Cancerología	1946	Cancer	INCan	Never
Instituto Nacional de Neurología y Neurocirugía				
*Manuel Velasco Suárez*	1952	Neurology	INNNMVS	2012–2017
Instituto Nacional de Enfermedades Respiratorias				
*Ismael Cosio Villegas*	1969	Neumology	INER	Never
Instituto Nacional de Pediatría	1970	Pediatrics	INPed	1997–2000
Instituto Nacional de Perinatología		Gynecology, obstetric,		
*Isidro Espinosa de los Reyes*	1977	and neonatology	INPer	Never
Instituto Nacional de Psiquiatría				
*Ramón de la Fuente Muñiz*	1979	Psychiatry	INPsi	2009–to date
Instituto Nacional de Salud Pública	1987	Public health	INSP	Never
Instituto Nacional de Médicina Genómica	2004	Genetics	INMEGEN	Never
Instituto Nacional de Rehabilitación		Rehabilitation		
Luis Guillermo Ibarra Ibarra	2005	and orthopedics	INR	Never
Instituto Nacional de Geriatría	2008	Geriatrics	INGer	Never

The MNIHs are decentralized organizations of the federal public administration, with their own legal personality and resources, grouped within the governmental health sector. Their main purpose is the provision of high-specialty medical care for population—in particular those with very low income, or challenging disease conditions—the clinical and basic scientific research within their medical specialty scope, and the training in both medical care and research (clinical and basic) of highly qualified human resources (physicians, nurses, biologists, social workers, chemists, etc.). Their scope of action covers the entire national territory [23].

Within the MNIHs system, there is a lack of evidence regarding the quantitative representation of the gender imbalance in executive management positions and wage gaps (average monthly net pay).

Therefore, the objective of this article is to analyze the situation of the MNIHs regarding gender imbalance, both in terms of quantitative representation in executive management positions and wage gap measured as the average monthly net pay, and discuss existing differences as framed in relation to the Mexican healthcare context.

## Methods

### Data collection

A local database was built using the available data from the National Transparency Obligations portal, which was manually collected [24]. The database contained the following recorded variables: average monthly net pay, gender, executive management positions (not operative level), and MNIH name as described below.

The average monthly net pay is defined as the money spare after deductions from the brute wage, and it is expressed in US dollars (around 18.85 MXN per 1 USD). It is basically composed of the regular wage plus various monetary compensations such as special allowances, assistance for updating expenses, social security, assistance for pantry, and transportation. Gender is a three valued field, according to the person actually in functions, i.e., man, woman, or vacant. Executive management positions are the hierarchical job position descriptor within each MNIH: Managing Director, Deputy General Manager, Area Director, Deputy Director, Department Head B, Department Head A, and Department Head. Finally, all thirteen MNIHs were included. Hereafter, we will use the Spanish names and acronyms of the corresponding MNIHs (see Table 1).

### Visualization

In order to illustrate the gender imbalance distribution in the executive management positions within and between the MNIHs, a multidimensional circular visualization was built using circlize [25], an R implementation of the Circos plot approach. This plot type is an efficient tool not only for data visualization, but also to unblur relationships between elements, since it intuitively depicts data over multiple tracks focusing on the same object [26].

### Statistical analyses

The average monthly net pay was modeled using a generalized linear model (glm) with gamma distribution, inverse link function, and linear predictor including gender, executive management positions, MNIH, and all the possible double plus the only triple interaction available. The vacant job positions were excluded from the analysis, given the fact that they are just a transition present in the snapshot taken from the data acquisition. The R-based glm implementation of the Hastie and Pregibon algorithm was used in this work to fit the data as specified by InfoStat software version 2018 [27, 28]. The model goodness of fit was assessed using analysis of variance (ANOVA) with a type III sum of squares. Posterior Fisher’s least significance difference (LSD) was obtained using Bonferroni’s multiple test correction. The significance threshold was set to *α*=0.05 for all statistical tests.

## Results

### Circular visualization

**Fig. 1:**
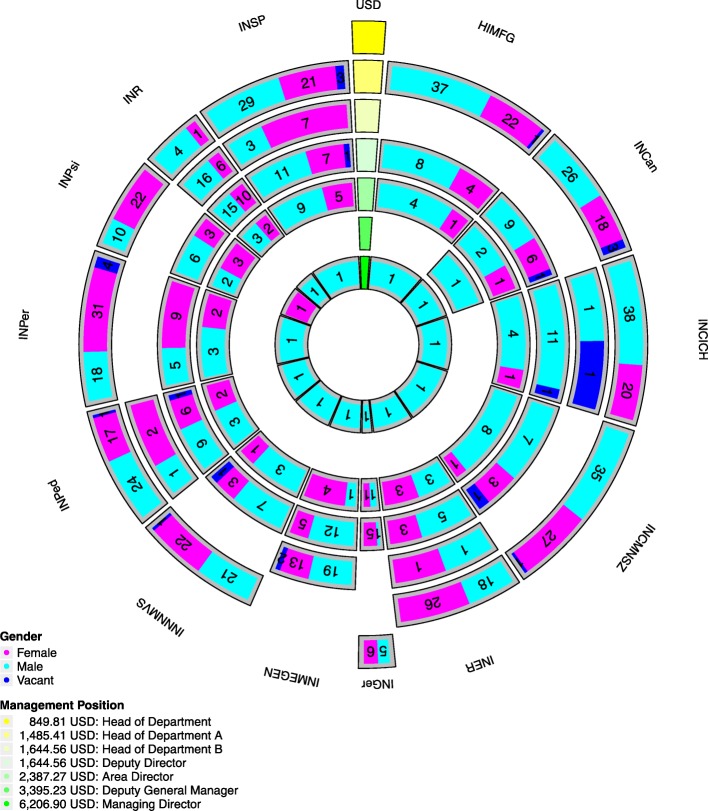
Multidimensional circular visualization. INR, Instituto Nacional de Rehabilitación; INSP, Instituto Nacional de Salud Pública; HMFG, Hospital Infantil de México Federico Gómez; INCan, Instituto Nacional de Cancerología; INCICH, Instituto Nacional de Cardiología Ignacio Chávez; INCMNSZ, Instituto Nacional de Ciencias Médicas y Nutrición Salvador Zubirán; INER, Instituto Nacional de Enfermedades Respiratorias; INGer, Instituto Nacional de Geriatría; INMEGEN, Instituto Nacional de Medicina Genómica; INNNMVS, Instituto Nacional de Neurología y Neurocirugía Manuel Velasco Suárez; INPed, Instituto Nacional de Pediatría; INPer, Instituto Nacional de Perinatología; INPsi, Instituto Nacional de Psiquiatría. The monthly average wage is expressed in US dollars

A multidimensional circular visualization of gender imbalance distribution of MNIH executive management positions is shown in Fig. 1. The results of the circular visualization will be described by tracks, sector, and colors as follows:

#### Representation by tracks

A track is defined as a concentric circular partition (ring). The circular layout depicted in Fig. 1 has seven tracks. Each track corresponds to an executive management position. They are organized in a hierarchical order, from the inside (highest) representing the Managing Director to the outside (lowest) belonging to the Department Head. In this context, the position with the largest occupancy number is the Head of Department A with 501 persons in total. On the contrary, the least occupied positions are Head of Department (11 persons) and Managing Director (13 persons).

#### Representation by sector

A sector is defined as a triangular partition. In Fig. 1, each sector refers to one of the given MNIHs. Here, the sector width stands for the proportion of leadership positions present in the MNIHs. The data gathered in our database shows that the MNIH with fewest executive management positions is the INGer (*n*=20, corresponding to the 2.4%), whereas the INSP (*n*=97 or 11.3%) and the INCNMSZ (*n*=84 or 9.9%) have four times more positions.

#### Representation by color

Sectors within the tracks are colored according to gender. Blue cells represent male-occupied positions, whereas pink cells are female-occupied offices. It is noticeable that cells in the outer tracks—which represent lower hierarchy management positions—are relatively gender balanced. Some institutions even show a slight female-biased imbalance (such is the case of INPer, INPsi, and INER). In stark contrast, is what happens at higher institutional leadership positions (inner tracks). This is best represented in the case of the Managing Director position, with a 12:1 male-to-female ratio. Specific details of all these ratios will be presented in the next subsection.

### Regression analysis

A generalized linear model was used to model the relationship of the monthly net pay versus gender, institute, executive management position, and their corresponding double and triple interactions as described in the “Methods” section. ANOVA results for the fitted model are presented in Table 2. The results show that for all the effects tested, but the triple interaction, there exists a statistical significant effect (gender, institute, positions, and double interactions). There are also significant differences between the interaction effect of gender and institutes, gender and positions, and institute and positions. However, there is no significant difference between the interaction effect of gender, institute, and positions.

**Table 2:** ANOVA results for the fitted model

Effect	SS	Df	*F* values	*P*r (> *F*)
Gender	0.01	1	12.23	< 0.001
Institute	0.43	12	41.69	< 0.001
Executive management position	3.75	3	1 438.72	< 0.001
Gender: institute	0.04	12	3.39	< 0.001
Gender: executive management position	0.01	2	5.57	0.004
Institute: executive management position	0.74	38	22.34	< 0.001
Gender: institute: executive management position	0.03	25	1.17	0.254
Residuals	0.63	725	NA	NA

*SS* type III sum of squares, *Df* degree of freedom, *F values* the value of the two-way ANOVA *F* statistic, *Pr (> F)* the *P* value associated with rejecting the null

hypothesis of equal means, *NA* does not apply

**Fig. 2:**
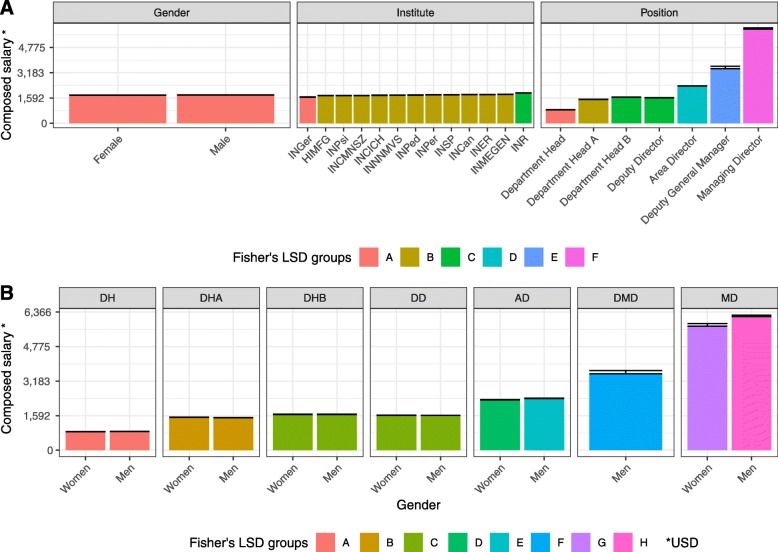
Posterior model test results for monthly net pay. Fisher’s least significance difference (LSD) was obtained for the main effects (**a**) and for the executive management position by gender double interaction (**b**). Fisher’s LSD letter groups are color coded for the corresponding effects levels, where different letters/colors represent statistically differences over Bonferroni adjusted *P* values (Bonferroni *P* < 0.05). INR, Instituto Nacional de Rehabilitación; INSP, Instituto Nacional de Salud Pública; HMFG, Hospital Infantil de México Federico Gomez; INCan, Instituto Nacional de Cancerología; INCICH, Instituto Nacional de Cardiología Ignacio Chávez; INCMNSZ, Instituto Nacional de Ciencias Médicas y Nutrición Salvador Zubirán; INER, Instituto Nacional de Enfermedades Respiratorias; INGer, Instituto Nacional de Geriatría; INMEGEN, Instituto Nacional de Medicina Genómica; INNNMVS, Instituto Nacional de Neurología y Neurocirugía Manuel Velasco Suárez; INPed, Instituto Nacional de Pediatría; INPer, Instituto Nacional de Perinatología; INPsi, Instituto Nacional de Psiquiatría; DH, Department Head; DHA, Department Head A; DHB, Department Head B; DD, Deputy Director; AD, Area Director; DMD, Deputy Manager Director; MD, Managing Director

Posterior Fishers’ LSD test results are presented in Fig. 2. In Fig. 2a, each panel describes the fitted monthly net pay for each main effect (gender, institute, and executive management position) versus the corresponding levels. The bar color represents Fisher’s LSD groups (A–F) of each factor level present in the tested main effect, if any, where different letters/colors represent statistical differences over Bonferroni adjusted *P* values (Bonferroni *P* < 0.05).

However, there are three monthly net pay institute steps: (i) the INGer in the lowest ladder level, (ii) the majority of the remaining institutes in the middle, and (iii) the INR at the highest step. As regards the executive management position panel, the monthly net pay is in a kind of exponential fashion, where the former position has a lower wage except for “Department Head B” and “Deputy Director” where both belong to the “C” (green) group.

In order to further explore how the significant effect of executive management position impacts in the monthly net pay by gender, the corresponding Fisher’s LSD test was applied as depicted in Fig. 2b. As a matter of fact, the observed pattern of executive management position panel of Fig. 2a holds, but there are four additional points to mention: (i) No gender differences are found in Department Head, Head A/B, or Deputy Director. (ii) In both Area Director and Managing Director, men have higher monthly net pay than women. (iii) The interaction effect for the monthly net pay could not be assessed for the Deputy General Manager, as there exists only a man at INCan.

These results showed that there is a gender imbalance distribution among the hierarchical position, for example, Managing Director represent only 1.6% of the total management positions and only one woman had access to this position (7%). In contrast, the Head of Department A position represents 57% of the total managerial position and 46.6% are carpeted by women.

## Discussion

Gender hierarchy in the healthcare professions is a complex sociological phenomenon. For decades, it has been argued that medicine is a patriarchal enterprise headed by men to maintain women in a subordinate position. According to these theories, men have consistently used gender exclusionary strategies to maintain a male monopoly [29]. Then, during the 1920s and 1930s, aspiring women doctors increased their participation in male-dominated professions [30, 31]. Since the 1970s, the numbers of women in medicine have increased significantly around the world [32].

It is well documented that even if women’s awareness of the importance of their active participation in healthcare professions is growing, they still struggle with the lag of many to accept them, which leads to obstacles to access and promotion to leadership positions and to the training needed to qualify for this access, as well as to an already mentioned inequality in working conditions and wages [33–39]. Such disparity is more pronounced in academic medicine, especially in the cardiac and surgical specialties (i.e., in high-paying specialties) [40, 41].

Studies on gender differences about preferences, attitudes, and skills are often contradictory. It is indeed difficult to assess the reasons why leadership positions are assumed in some health institutions. The most common are believed to be mainly related to personality, character, labor merits, social and political relations, and prestige, as well as to academic and administrative inclinations [42].

The concept of self-efficacy to leadership offers a potential resolution of this contradiction. Various theories argue that effective leadership is the result of the appropriateness or fit between particular behaviors (being someone well trained and willing) and particular situations (being in an appropriate and welcoming context) to the functional necessities of each leadership organization. Hence, instead of placing barriers to high-level training, organizations must design and include effective policies to prepare and encourage women’s leadership if they want to be inclusive [37, 43, 44].

In Mexico, command posts in hospitals, health ministries, universities, or medical academies have been in charge of men for hundreds of years already. These higher management and director positions require full-time dedication and have often been filled by 50 plus-year-old men. This could be explained by the fact that the feminization of medicine in Mexico has been a recent phenomenon so that time has not allowed for women’s leadership to become common [42].

If we analyze the Area Director position and the size of each MNIH, we found that in the INGer, which is the smallest institute with only 19 (2.4%) executive managers, woman and man are found in the same proportion, while the largest institutes, like INSP or INCMNSZ, showed an apparent gender gap; for example, in the INSP, 36% are women and in INCMNSZ only 11%.

On the other hand, in the Deputy Director position, gender gap is more evident and could be related to antiquity of MNIH. For example, in 10 of the 13 MNIHs (some of the oldest institutions), this position is occupied predominantly by men. In the INCICH (the eldest of all), historically, women have never been represented in this position, while in two more, INger and INPer are occupied by women more frequently.

In this research, we found that executive positions in MNIHs are significantly lower for female than those of male executives and that the wage gap exists mainly in the lower ranks of the hierarchical order, where women are relatively highly concentrated. This phenomenon has already been reported in other cases [45].

Consistent with our finding, other studies have shown that while the majority of the healthcare workforce is female, women are still underrepresented in healthcare leadership positions [46–49]. Women represent 70 to 80% of the total of the workforce; however, only 24% reach senior executive positions, and furthermore, only 18% of women occupied general manager positions in hospitals [50, 51].

Another example of this situation is the health boards, where women represent only 14% of the population [52]. This is the case of the World Health Assembly, where women occupied a quarter of the Managing Director position, even though they represent three quarters of the total health workforce [3].

In our results, the gender imbalance in executive management positions apparently was not affected by temporal changes; however, other studies have demonstrated that temporality or antiquity could determine the organizational policy and greater access to the leadership position, especially those determined by the legal environment [53].

A number of traditional and critical theories have been put forward by various institutions, organizations, authors, scholars, researchers, and development practitioners, somehow, to explain or contextualize the issue of gender equality and lower female empowerment.

In the twentieth century, perspectives given by sociological research and gender studies have provided some relevant frameworks aimed to explain why women are underrepresented in executive management positions [54].

In what follows, we will present and discuss some of these:

*Inequality theories*, the apparent universal subordination of females over the centuries (supposedly a biological explanation): *Structural functionalism* argues that gender roles were established aimed at keeping the division of labor and family system functioning properly because men typically took care of responsibilities outside of the home, and women typically took care of domestic responsibilities [54].

According to the *Conflict theory*, society is driven by a struggle for dominance among social groups, men as the dominant group and women as the subordinate group, and the social problems are created when dominant groups exploit or oppress subordinate groups. *Feminist theory* considers that the patriarchal family is realized by perpetuating male dominance. *Symbolic interactionism* aims to understand human interaction by analyzing the created behavior related to the interpretation of masculinity or femininity characteristics which may help us understand societal labeling, institutionalized sexism, and gender disparities [54].

Recently, gender theories have received a great deal of attention, in particular the ones dealing with gender barriers and disparities in the corporate suite. *Androgyny theory*, or feminization of management, states that females and males could possess both masculine and feminine traits. *Gender stereotyping theory* states that managerial roles should be masculine occupations or in any case reserved for older females. The perpetration of such ideas from the corporate world does not seem far from what happens at higher managerial and directive positions in healthcare systems [55].

**Fig. 3:**
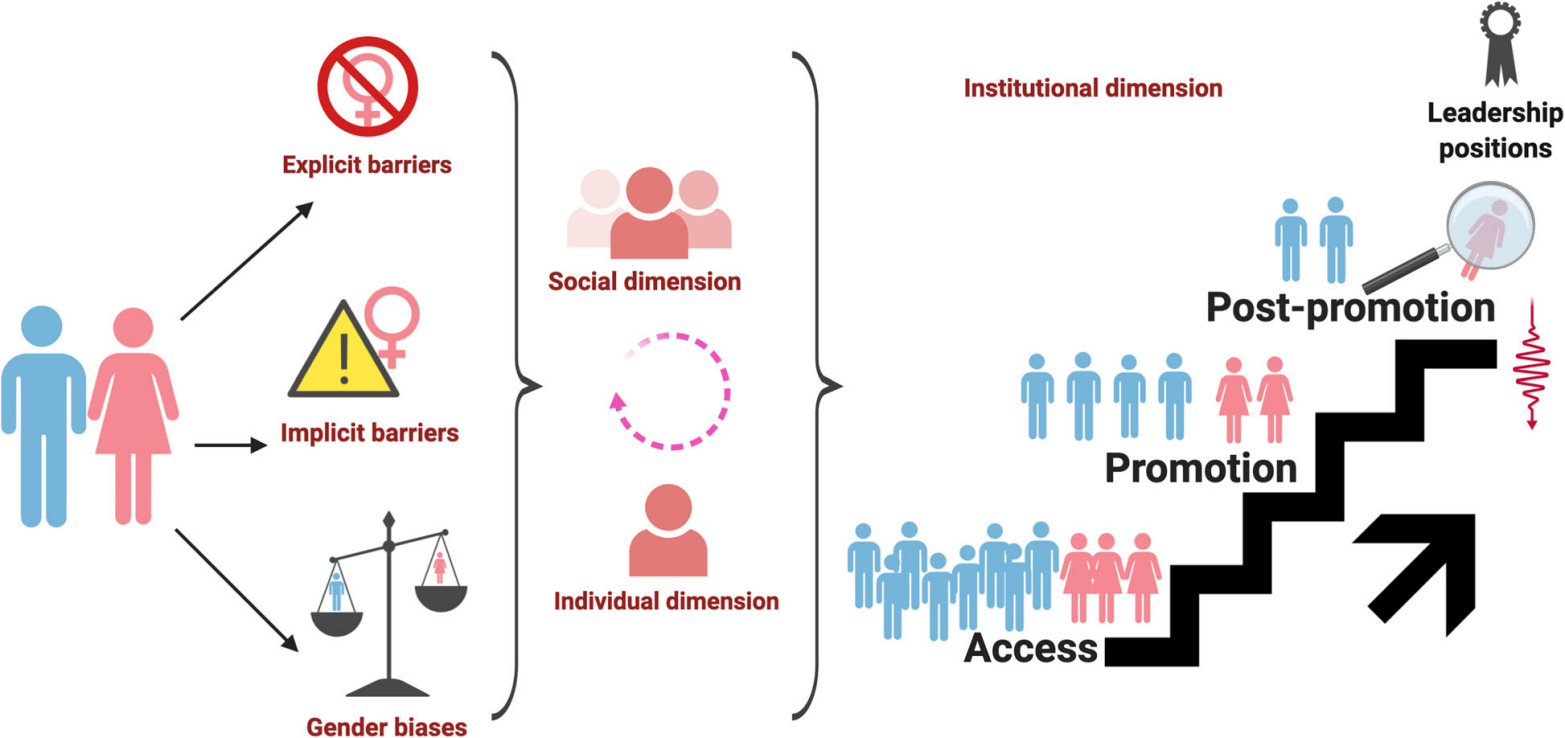
Conceptual model of structural of multilevel gender barriers. Representation of stereotype-based cognitive barriers in a social dimension, individual dimension, and institutional dimension

*Expectation states theory* argues that gender leadership style is deeply embedded in status, beliefs, and organization’s social hierarchy because of the rules of the gender system. Also, advantaged groups (as males) are seen in society as having greater competence and social significance than disadvantaged groups (as females); this belief, in turn, becomes grounded in inequalities. *Role congruity theory* takes into account an individual’s gender role, its congruity with other roles [55]. Well-documented more specific theories, related to stereotype-based cognitive bias, social dimension, individual dimension, and institutional dimension, explain the barriers and various contextual factors that impede women’s opportunities for leadership positions in work organizations. Figure 3 shows a conceptual model of structural of multilevel gender barriers.

Stereotype-based cognitive bias may take two forms:

*Explicit barriers* are related to clear personal beliefs about women, such as believing that women are less committed to their careers than men and believing that women are worse leaders than men. Women also encounter additional explicit hurdles in becoming leaders, and they face a double bias by the reason of motherhood or by the color of skin [56].

*Implicit barriers* refer to unconscious stereotypes or attitudes which are harder to see, but they still influence judgment and actions towards women. The stereotype-based perspective towards women has been used to explain their supposed lack of fit for leadership roles in medicine. These stereotypes center on perceived characteristics, skills, and aspirations of women and how they have been perceived to not coincide with what it has been declared valuable for effective leadership, e.g., “how can a woman be the leader of a large hospital?” the challenges can be “too much” for her [39, 57, 58].

Contemporary theories of gender as a multilevel or structural system also explain the problem in terms of additional dimensions, as follows:

*Social dimension (homosocial reproduction)* suggests that decision-makers tend to appoint leaders like themselves in terms of gender, race, age, and background; in-group favoritism leads men to prefer other men for high-level promotions and appointments. Hence, male directives will train and elect other males to succeed them. Social hierarchies are hence part of the reason why the so-called glass ceiling phenomenon exists [37, 38, 55].

*Individual dimension* suggests that women face significant limitations on their individual ability to display typical leadership qualities. To counter this burden, some women have de-emphasized her gender status when interacting with male colleagues, training to be assertive, driven and/or competitive in spirit, without failing to emote more stereotypically feminine qualities such as warmth and passivity [37, 59].

*Institutional dimension* includes some processes that take place within the workplace that can limit the access, recruitment, retention, promotion, and advance of women to leadership positions. Also, these mechanisms are well reported in the literature termed as the glass cliff, the decision-maker diversity, and the savior effect [44].

Also, by looking beyond individual actors and behaviors, a structural sexism perspective highlights the discriminatory character of institutional arrangements. Let us consider some possible mechanisms that may be present in healthcare systems [60].

Barriers related to the access and recruiting are as follows:

*Gender-segregated social and professional networks*. It has been discussed that men are more likely than women to have access to more abundant and timelier information about job openings, which gives potential female applicants a handicapped position since they may lack critical information about job availability relative to their male counterparts [44].

*In-group preferences*. Gender stereotypes strongly influence decision-makers’ evaluations of job candidates, in contexts where traits required for performance tend to be stereotypically masculine. This ideas are related to those of homosocial reproduction theories, already mentioned [44].

Barriers related to the retention and promotion are as follows:

*Glass ceiling phenomenon* refers to an invisible barrier to women’s promotion to top positions by means of other implicit mechanisms, such as lack of quality mentoring, strong professional networks, social ties to elites, workplace support, and insider information [37, 38].

*Glass cliffs scenario* suggests that women will be appointed to leadership in poorly performing firms that are struggling, in crisis, or at risk to fail because they are viewed as superior interpersonal managers and more capable of taking the blame for failure. As a result, women leaders often experience shorter tenures, and again, the men associates are the ones perceived as ideal managers for successful firms [37].

*Labyrinth theory* suggests that women and men use different routes to climb the corporate ladder. Women break impermeable barriers in small numbers, such as in a labyrinth. The labyrinth represents the possible multiple routes that women follow in their career, because routes do exist for females, they are just not direct routes, but entangled ones [55].

Barriers related to the advance and postpromotion are as follows:

*Token status* suggests it is believed that those who comprise less than 15% of a group’s total are expected to experience a variety of hardships in the workplace, leading to weaker social and professional networks; reduced organizational support, information, and assistance from peers and subordinates; heightened visibility and scrutiny; exaggerated stereotypes; and exclusion and isolation. As a result, token women leaders often experience reduced performance and lower job satisfaction [37, 38, 55].

*Role incongruity theory* suggests that the association of leadership with masculinity motivates assumptions about the type of person who can successfully fulfill leadership roles, leading to negative evaluations of women, irrespective of their preparation, ability, or performance [38].

On a related scenario, the so-called savior effect predicts that women will be granted less of opportunities to prove their leadership capabilities within a large organization, so in that cause, they are likely to be replaced by more traditional men leaders who will be brought in to save the firm apparently from poor leadership [37].

Perhaps, the gender gap could be explained by at least two phenomena: the so-called glass ceiling, which is used as a metaphor for the invisible barrier that veiled the advance of women towards higher ranks within the organizations [61, 62], and the implicit bias, which is based on personal believes that act based on unconscious internalized schemes and therefore often act through discriminatory unconscious participants’ behaviors [63, 64].

Other forms of discrimination are wage gap between genders, explained by different working hours or different occupations. There was an analysis based on median wages from the Labour Force Survey data compiled by the International Labour Organization (2000–2018, over 104 countries) where 21 countries showed an average gender pay gap of around 28% exists in the health workforce [51]. In working sectors overpowered by women, work is often undervalued and underpaid; this is the case of the health sector where high-income countries have 26% wage gap and is even higher in middle-income countries 29% [50].

Interestingly, in our case, we found no evidence of the wage gap between genders since gross salaries in the Mexican government is by law fixed for a given position, regardless of the gender or any other characteristic of the position holder; therefore, women in executive management position are remunerated at similar levels to their male counterparts. The only case in which we detected a wage gap was in the Managing Director position (Fig. 2b).

These results are consistent with what Cohen and Huffman reported about female managers associated with lower gender wage gaps, especially when those women were in managerial positions of relatively high status [53]. However, other studies indicated that in many institutions worldwide, women are still extremely underrepresented in leadership positions and also have access to lower salaries on average than their male peers [64–67].

Other work also reported that a wage gap between men and women (19% less) in leadership positions has been persistent. Additionally, it was noted that women tended to work in specialized areas that are not the usual professional rates for executive leadership positions such as nursing services, planning, marketing, and quality assurance [68, 69].

Strong positive effects have been reported, both on the size of the workplace and in the rise of gender integration [53]. Women underrepresentation in some fields of medicine is critical, because lack of gender diversity in the medical workforce can modify sex-specific aspects, such as the ones related to gender unbalanced financial coverage, patient care, and outcomes [40].

Whether or not this situation has given place to the establishment and perpetuation of gender-based healthcare inequalities is still uncertain. What is not in doubt is that many of these men-designed policies are indeed creating unequal treatment condition of healthcare available to women. One instance that has been recently addressed by our group is related to gender inequalities in public healthcare insurance coverage of myocardial infarction in women [70]. The current situation covers up healthcare security up until 60 years old, both in men and women cases. However, it is well known from social epidemiology studies on myocardial infarctions that women are prone to suffer from cardiac failures later in life (postmenopause), often long after the age of 60 years old [71]. This known fact has been however overlooked by the mostly men-driven public policy-making bodies. Is this inequality a consequence of gender imbalanced directive counsels, we do not know.

The influence of female medical directive decisions on Mexican healthcare system is special. In recent years (since 1997), out of 13 MINHs, only three Mexican leaders have reached the general direction: Dr. Alessandra Carnevale Cantoni, MD, at INPed (1997–2000); Dr. Teresa Corona Vázquez, MD/PhD, at INNN (2012–2017); and Dr. María Elena Medina Mora, PhD, at INPsi (2009–to date), and for the first time, a woman headed the highest authority in the Secretary of Health in Mexico, Dr. Mercedes Juan López, MD (2012–2016). To add some perspective, there have been up to 85 opportunities to reach the general director status along these years and only three of these have resulted in a woman being elected [42, 72].

There is no doubt that gender differences have been critical in making different decisions about medical care, with a gender perspective, as well as the access and financing process in some areas of the institutional health system. For example, multidisciplinary research with a gender perspective in neuropsychiatric disorders, as well as the study of addictions in Mexican women, has been favored scientific evidence to financing practices for access and care, and has contributed to the formulation of emerging gender and health policies, but also has promoted a greater number of women in decision-making positions and gender professionalization [73–75].

With this in mind, the vision of the women Mexican health administration, during the 2012–2018 period, was to transform the Mexican healthcare system so that it ensures equitable and effective access to quality healthcare [76]. Within its management, the National Observatory of Health Inequities (NOHI) was formed, and recently, NOHI published its first report on health gaps and differences in access. NOHI has been also proposed to generate knowledge that will allow the Mexican government and its Ministry of Health to achieve a unique, public, free, and egalitarian system. The years to come will witness the efficacy of these relevant actions in the practice [77, 78].

In spite of their large organizational size, there is no evidence of size integrating effects at the MNIHs, as pointed out by our results. In the case of healthcare, occupational segregation still persists with strong disparities on leadership positions, salaries, and working conditions. Most of the healthcare workforce is feminine, and women’s participation in health-related areas is consistently higher than in the bulk of science. However, feminization of the workforce happens in an unequal manner: around 75% of the worldwide healthcare workforce is feminine, but women are disproportionately relegated to the lower cadres of health work [79]. As already discussed, this is the case of the MNIHs supported by our results.

According to the High-Level Commission on Health Employment and Economic Growth, gender biases in the healthcare sector undermine inclusive economic growth, employment, appropriate work conditions, and the achievement of gender equality. There is a consensus that gender equality in science, medicine, and global health has the potential to lead to important health, social, and economic benefits [79]. One goal of the present study is, thus, to provide policy boards and decision-makers with carefully analyzed data to help them make informed choice on these issues.

In August 2018, the president of the World Bank pointed out that human capital, the potential of individuals, will be the most important long-term investment that any country can make to the prosperity and quality of life of people in the future. However, the chronic lack of recruitment, promotion, and retention of women in science and medicine is due to systemic, structural, organizational, institutional, cultural, and social barriers to equity and inclusion. Such barriers need to be dismantled in order to ensure optimal development of the said human capital [11].

Gender imbalance is pervasive in many contexts within medical leadership, but some authors have stated that once uncovered, such imbalance may become malleable. They sustain that possible strategies include a longitudinal approach combined with progressive small-scale training that will be more effective to empower women and may result in durable change towards an increasingly modern and developed society, one with a narrower gender gap [55, 58].

Multidimensional policies in the institutional structures are needed to attain these goals. Gender diversity, particularly the presence of influential women among decision-making ranks, can also impact women’s mobility and tenure, as well as help reduce the impact of gender homophily and token status [37, 44, 55, 80].

It has been argued that annual salary and labor condition reviews with a gender focus, as well as implementation and frequent monitoring systems for harassment and discrimination, can help women not only to survive, but to thrive in the medical leadership field [56].

Socialization processes may improve mentorship, guidance on career goals, and professional advancement of women. Inclusion on social networks to be well informed of job opportunities is also critical [44, 55, 56].

Derived from the previous cases, it is proposed that in order to reduce gender imbalance in the Mexican case, it is critical to design structural policies for access and promotion of equitable leadership positions, based on institutional, cultural, social, and biological differences. Additionally, it is proposed to generate future studies, based on multilevel frameworks for research on the occupational situation, availability of resources, and salaries of health personnel.

## Conclusions

Although, around the world, a large amount of support has emerged to give visibility and help combat gender inequalities for women in science, medicine, and health, there is still a lack of gender parity in leadership positions, despite the fact that women constitute a large part of the global health workforce.

Unfortunately, our results led us to conclude that it continues to be a relevant underrepresentation of women in formal positions of high-level medical leadership, as pointed out by our analysis of the case of the Mexican National Institutes of Health. The situation seems to be even more worrying since there is a perception that other healthcare institutions in Mexico are even further behind in terms of inclusion and narrowing of the gender gap. A vigorous and consistent monitoring of top hospital management in Mexico is thus required. Our results have suggestive political implications. We hope that our findings will motivate further studies on the role of organizational change in shaping the contours of inequality in the workplace.

## Data Availability

The datasets used and/or analyzed during the current study are available from the corresponding author on reasonable request.

## Abbreviations

AD: Area Director
ANOVA: Analysis of variance
glm: Generalized linear model
INR: Instituto Nacional de Rehabilitación
INSP: Instituto Nacional de Salud Pública
HMFG: Hospital Infantil de México Federico Gómez
INCan: Instituto Nacional de Cancerología
INCICH: Instituto Nacional de Cardiología Ignacio Chávez
INCMNSZ: Instituto Nacional de Ciencias Médicas y Nutrición Salvador Zubirán
INEGI: Instituto Nacional de Estadística, Geografía e Informática
INER: Instituto Nacional de Enfermedades Respiratorias
INGer: Instituto Nacional de Geriatría
INMEGEN: Instituto Nacional de Medicina Genómica
INNNMVS: Instituto Nacional de Neurología y Neurocirugía Manuel Velasco Suárez
INPed: Instituto Nacional de Pediatría
INPer: Instituto Nacional de Perinatología
INPsi: Instituto Nacional de Psiquiatría
DH: Department Head
DHA: Department Head A
DHB: Department Head B
DD: Deputy Director
Df: Degree of freedom
DMD: Deputy Manager Director
LSD: Least significance difference
MD: Managing Director
MNIHs: Mexican National Institutes of Health
MNIH: Mexican National Institute of Health
MXN: Mexican pesos
NA: Does not apply
OECD: Organization for Economic Cooperation and Development
SS: Type III sum of squares
SSA: Secretaria de Salud
STEM: Science, technology, engineering, and mathematics
USD: American dollars
